# Uterine Position and Diagnostic Accuracy of Transvaginal 2D, 3D, Transrectal and Transabdominal Ultrasonography in the Assessment of Endometrial and Uterine Cavity Abnormalities—A Narrative Review

**DOI:** 10.3390/jcm15155978

**Published:** 2026-07-31

**Authors:** Małgorzata Satora, Julia Ochocka, Julia Janowska, Lucja Zaborowska, Artur Ludwin

**Affiliations:** 1 1st Department of Obstetrics and Gynecology, Medical University of Warsaw, 02-015 Warsaw, Poland; 2 Doctoral School, Medical University of Warsaw, 02-091 Warsaw, Poland

**Keywords:** transvaginal ultrasound, transabdominal ultrasound, transrectal ultrasound, two-dimensional transvaginal ultrasound, three-dimensional transvaginal ultrasound, anteverted uterus, retroverteduterus, axial uterus, uterine position, endometrium

## Abstract

Transvaginal ultrasound (TVS) remains the most frequently performed examination in gynecological patients to evaluate the uterine cavity. Visualization of the endometrium may be limited due to certain uterine positions, particularly axial and retroverted orientations. Alternative diagnostic methods, such as transabdominal ultrasound (TAS) and transrectal ultrasound (TRS), may be useful in patients in whom visualization is suboptimal using the TVS. The aim of the study was to analyze the literature regarding the diagnostic performance of TVS, TAS, and TRS in detecting uterine cavity abnormalities in women with various uterine positions. Studies evaluating ultrasound assessment of uterine cavity pathologies with reference to uterine position were included in this review. The available research suggests that uterine position may influence ultrasound visualization. TAS has been shown to demonstrate lower diagnostic reliability in retroverted or axial uteri, particularly in patients with obesity or when focal lesions are present. TRS may provide improved assessment of endometrial thickness in patients with axial uteri compared with TVS. Uterine position may be a factor affecting the accuracy of evaluation of the uterine cavity in ultrasound. Awareness of these limitations and the use of additional imaging modalities may improve diagnostic accuracy in selected patients. Further studies are needed.

## 1. Introduction

Despite continuous medical advancements, transvaginal ultrasound (TVS) remains one of the most frequently performed examinations in gynecological patients. Its widespread use is primarily attributable to its ease of performance in the office setting, low cost, and, most importantly, its non-invasive nature [1]. Despite its numerous advantages and clinical benefits, the diagnosis of endometrial and uterine cavity lesions in many women should not solely rely on this modality. Several limitations may limit adequate uterine visualization; these include uterine fibroids, endometriosis, or an imperforate hymen [2]. Interestingly, currently available literature indicates that the position of the uterus may also influence the imaging of the endometrium and uterine lesions with TVS. Adequate visualization of the uterine cavity may be particularly challenging in cases of an axial uterus or retroverted uterus. In such situations, the endometrial stripe may be aligned parallel to the TVS beam [3,4]. This contrasts with imaging in the case of an anteverted uterus, where the ultrasound beam is perpendicular to the endometrial axis, which facilitates imaging [5].

When visualization of endometrial lesions is difficult, the International Endometrial Tumor Analysis recommends saline contrast sonohysterography (SCSH) [6]. However, this method is not routinely available in every practice and may be associated with a number of complications. Finally, this examination may be painful in women with vulvar and vaginal atrophy [7]. Therefore, there is a need for alternative diagnostic tests that could allow for a satisfactory assessment of the endometrium and uterine changes in a selected group of patients. In routine practice, the most commonly used modalities besides TVS are transabdominal ultrasound (TAS) and transrectal ultrasound (TRS) [8]. Uterine position may substantially influence the quality of visualization obtained using TVS, TAS and TRS. To our knowledge, no studies have compared these three approaches simultaneously.

In this narrative review, the authors summarize the current literature regarding the role of TVS, TAS and TRS in the detection and differentiation of uterine cavity pathologies in women with a uterus in various anatomical positions.

## 2. Materials and Methods

A literature search was performed in February 2026. No restrictions on publication year were applied during the literature search. The studies included in this review were identified from the PubMed, Scopus, Embase, Web of Science, and Cochrane databases. The search strategy was developed using combinations of terms related to the assessment of uterine position, endometrium and uterine cavity with ultrasound. We used keywords: transvaginal ultrasound, transvaginal ultrasonography, two-dimensional transvaginal ultrasound, three-dimensional transvaginal ultrasound, transabdominal ultrasound, transabdominal ultrasonography, transrectal ultrasound, transrectal ultrasonography, endometrium, uterine cavity, endometrial pathology, uterine pathology, anteverted uterus, retroverted uterus, axial uterus, uterine position, uterine orientation. The search was performed simultaneously by three authors (MS, JO, JJ). All possible discrepancies were resolved by a fourth author (AL). The search strings were modified according to each database’s model. There were no language restrictions, although the authors utilized English keywords.

Our aim was to create a narrative review; however, we used a paper selection to find appropriate articles. All articles were initially screened for the title and abstract. The inclusion criteria were studies evaluating ultrasound in uterine cavity pathology assessment according to uterine position. Conference abstracts, reviews, systematic reviews, technical reports, editorials, and letters to the editor were excluded from the analysis. We identified additional studies by contacting authors or experts, looking through all the articles that cite the papers included in the review (“snowballing” or forward citation searching), reference list checking (backward citation searching), and searching trial or study registers. 

## 3. Uterine Position

Uterine orientation within the pelvic cavity is a highly individual characteristic determined by the spatial relationship between the uterine body, the cervix, and the vaginal axis [9]. In anatomical terminology, it is essential to distinguish between two fundamental geometric parameters—flexion and version. Flexion is the angular relationship between the uterine body and the long axis of the cervix. Version is the angle between the long axis of the entire uterus and the long axis of the vagina. Anteversion is the most common position occurring among gynecological patients, observed in approximately 80% of women [10]. In this arrangement, the uterus is tilted anteriorly toward the urinary bladder. Retroversion, where the uterus is tilted posteriorly toward the rectum, occurs in approximately 4–20% of patients [11,12,13]. Furthermore, retroversion is much more common in the urogynecological patient population (34%), which strongly correlates with a 4.5-fold increased risk of significant uterine prolapse (grades 2–4) [14]. 

While the majority of uteri are anteflexed, version and flexion often occur in various combinations, creating various anatomical configurations. A uterus may be either anteverted and retroflexed or retroverted and anteflexed [10]. These unusual curvatures pose significant challenges during intrauterine procedures. Major retroflexion or such atypical uterine positions significantly increases the risk of iatrogenic uterine perforation if the operator fails to acknowledge the unexpected course of the uterine cavity [14]. Particularly during intrauterine device (IUD) insertion, embryo transfer, or hysteroscopic maneuvers, the risk of injury is higher unless the instrument’s path is correlated with the patient’s individual angles of version and flexion [15,16].

**Figure 1:**
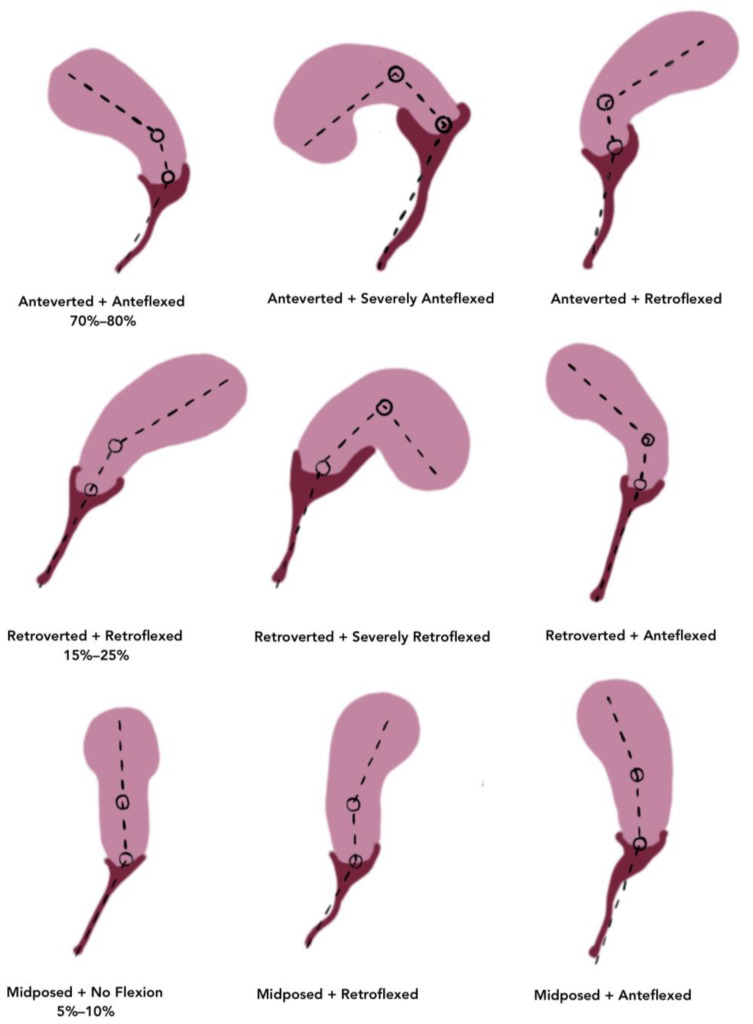
Sagittal schematic representation of different anatomical positions of the uterus in relation to surrounding pelvic structures. The urinary bladder is located anteriorly (left) and the rectum posteriorly (right). Dashed lines illustrate the vaginal, cervical, and uterine body (corpus) axes used to determine uterine version and flexion. Approximate prevalence estimates are based on published epidemiological studies of uterine position and may vary according to the study population, parity, menopausal status, and classification criteria [3,10,11,13].

Another anatomical variant is the axial uterus, which occurs when the angle of both version and flexion comes close to 180 degrees, resulting in a nearly straight alignment that follows the vaginal axis [13]. This position appears in 5 to 10% of women [10], yet it is often considered more as a dynamic state or consequence of pelvic adhesions and conditions such as deep infiltrating endometriosis rather than a fixed anatomical trait. This disease frequently affects the uterosacral ligaments and the posterior pelvic compartment, resulting in dense fibrosis which can pull the uterus back and lock the uterus in this straight, rigid position [17,18]. Clinical observation shows that uterine orientation can change, for example between TAS and TVS views, with a study reporting up to 32% of uteri appear anteverted on TAS examination but retroverted during TVS [10]. Furthermore, the presence of a full rectum, full bladder, or both can affect pelvic anatomy, impairing diagnostic accuracy [13]. This leads to the realization that an axial uterus diagnosis can be dependent on specific imaging variables, or it can be a permanent anatomical position. The uterine positions are shown in Figure 1.

In conclusion, uterine position is a dynamic and complex characteristic that dictates the accuracy of gynecological imaging and the safety of surgical interventions. The high prevalence of retroversion in specific patient groups and the complex diagnosis of the axial position calls for a personalized approach to imaging, often requiring the complementary use of TVS with an empty bladder to clarify diagnostic uncertainties.

## 4. Transvaginal Ultrasound

TVS is an extensively used imaging method of the female pelvis. It is minimally invasive, cost-effective, and widely available in routine gynecological practice [1,19]. It allows for the visualization of female reproductive organs such as the ovaries, adnexa, uterus, including endometrium, cervix, and surrounding pelvic structures. Once the probe is inserted into the vagina, sagittal, oblique, and transverse planes can be obtained. This technique provides images of higher resolution as compared to TAS, as it reduces the distance between the probe located in the vaginal canal and the female reproductive organs. In contrast, during TAS examination, the beam can be weakened by the anterior abdominal wall [20].

Furthermore, TVS is a first-line investigation in detecting deep invasive endometriosis [21,22], diagnosing adenomyosis [23], uterine fibroids, assessing myometrial invasion in patients with endometrial cancer or in the evaluation of endometrium in women with postmenopausal bleeding (PMB) [1].

Nonetheless, TVS has several limitations, including obesity, extensive myomas, adenomyosis, or previous uterine surgeries [1,5]. Notably, another limitation may originate from the uterine position, specifically the retroverted or axial uterus [11,24]. Available literature shows the prevalence of the retroverted uterus ranging from 12% [10] to 18% [15], and for the axial uterus, the prevalence of 5% [21].

The sagittal plane of a uterus is routinely obtained during TVS and enables the assessment of endometrial thickness (ET) [1], a parameter of high importance among women with PMB, which allows for identifying patients at high risk of endometrial cancer [25]. This parameter should be measured perpendicularly to the long axis of the uterus. It is reasonable as the visualization is most efficient whenever the angle of insonation is at 90° [26]. However, adequate measurement of ET is not always achievable. In a retrospective analysis of 472 postmenopausal asymptomatic women who underwent the TVS, the visualization of endometrium and the measurement of ET were unattainable in 180 patients [5]. One of the principal reasons for this was the presence of the axial uterus in 50 of these women (27.8%), which made the visualization suboptimal. Authors emphasize that such conditions pose a relevant diagnostic difficulty in which the exclusion of endometrial pathology cannot be based solely on TVS but requires further investigation with SCSH or hysteroscopy [5]. It is consistent with previous research, which demonstrated that the endometrium of an axial uterus might be difficult to assess as the ultrasound beam is in the same axis as the endometrium and does not fall at a perpendicular angle to the endometrium [10]. To improve the viewing of the axial uterus, a multimodal sonographic approach is suggested in order to optimize the angle of insonation [27]. In a study analyzing the correlation of ET measurements with the risk of endometrial pathology, 1168 women with PMB underwent TVS. In 30 of 1168 (2.8%) patients, the measurement of endometrium could not be obtained due to difficulties in identifying the longitudinal echo line which would allow visualization of the double-layer endometrium [28]. Visualization of the endometrial echo can be impeded by certain uterine positions, such as the axial uterus [29].

Another example of the position of the uterus influencing the results of examination is presented in an earlier mentioned study in which 480 patients underwent both a TAS with a full bladder and TVS with the bladder emptied. The prevalence of a retroverted uterus using TAS was 13% (61 of 480) in comparison to TVS, which was 18% (86 of 480), indicating that a distended bladder can antevert the uterus, masking its natural position (*p* < 0.001) [13]. These results are supported by another analysis in which 32% of retroverted uteri were anteverted on TAS and retroverted using the vaginal probe [10].

In a retrospective study on the accuracy of TVS and its comparison to MRI in the diagnosis of retroflexion in patients with endometriosis, 123 women underwent a TVS and MRI scan prior to surgery for endometriosis. Although retroflexion is a physiologic variant of the uterus, it is related to adhesions in endometriosis and chronic retracting posterior deep endometriosis, which are prone to damage surrounding structures and can complicate the surgery [30]. Anteflexion was detected using MRI in 43 (35%) patients and in 71 (58%) using TVS. Retroflexion was found with MRI in 80 (65%) women, while in TVS it was found in 52 (42%) cases. Using the MRI as a reference in this study, TVS showed all cases of anteflexion detected by MRI and 28 (39%) cases of retroflexion misdiagnosed as anteflexion (the false-negative group). The patients were further divided into anteverted (67/123) and retroverted (56/123) groups using the MRI. All misdiagnoses of flexion (28) in TVS were found only in the anteverted group, whereas all flexions in the retroverted group were observed correctly. The authors highlight that the possible reason for these misdiagnoses was the probe placed in the anterior vaginal vault, pressing and shifting the cervix, which resulted in the impression of an “anteflexed” uterus instead of retroflexed. This is a limitation of TVS in the diagnosis of retroflexion in women with endometriosis, specifically in false-negative cases of anteverted retroflexed uteri [30]. Table 1 presents the main characteristics of studies assessing TVS regardless of the uterine position.

The visualization of the uterine cavity and the endometrium in most cases is adequate and optimal, yet can be affected by less frequent uterine positions, such as the axial uterus. Awareness of this limitation is crucial among gynecologists and sonographers as it can influence the results of examination. Furthermore, the assessment of uterine position might also depend on the preparation of the patient prior to examination in terms of an emptied or distended bladder, specifically in cases of retroversion [10,14]. Eventually, the manipulation of the vaginal probe influences the evaluation of uterine position. Taking into consideration all of the above, in some cases, it is important to incorporate additional imaging approaches to provide an assessment of high quality and adequacy and not base the diagnosis solely on TVS.

## 5. Three-Dimensional Transvaginal Ultrasound

A relatively newer imaging technique is the three-dimensional transvaginal ultrasound (3D-TVS), which complements the 2D approach [31]. It is not considered a routine gynecological investigation [32]. This method is based on the acquisition of ultrasonographic volumes of the uterus or endometrial cavity and further analysis of acquired data, enabling a multiplanar reconstruction [33]. As the ultrasound volume is an assembly of a series of sonographic images folded together, it is possible to view the 3D-TVS volume in multiple planes. The visualization of the coronal plane is a major factor differentiating it from the regular TVS. 3D-TVS remains low-cost, non-invasive, and differs from the 2D-TVS in the ability to store the acquired volume data digitally for later analysis after discharging the patient [33]. The coronal plane in 3D-TVS enables the simultaneous visualization of uterine cavity morphology, the relationships of endometrium and myometrium at the uterine fundus and the cornual angles [34,35].

Although 3D-TVS is not thought of as a compulsory examination, recent studies prove its high diagnostic value, specifically in congenital uterine anomalies [34], adenomyosis, intrauterine devices (IUDs) and intrauterine adhesions [35,36]. Available literature indicates that 3D-TVS can reach comparable or, in some circumstances, even superior results in specificity and/or sensitivity when compared to 2D-TVS. A prospective study involved 72 premenopausal women with a benign pelvic pathology examined with 2D-TVS and 3D-TVS who subsequently underwent an earlier scheduled hysterectomy. The prevalence of adenomyosis based on histopathological examination was 44.4% (32/72). The diagnostic accuracy of 2D-TVS and 3D-TVS in relation to histopathology results was, respectively, 83% and 89%; the sensitivity was 75% and 91%, while the specificity was 90% and 88%. Authors highlighted the importance of the coronal view of the uterus gained in the 3D-TVS, which allows for accurate assessment of the Junctional Zone (JZ), a crucial structure in the diagnosis of adenomyosis [36]. Similarly, in the diagnosis of adenomyosis of the inner myometrium in 110 premenopausal women presenting with abnormal uterine bleeding (AUB), the sensitivity for 3D-TVS and 2D-TVS reached 69% and 72%, respectively; while for specificity it was 86% and 76% [37]. An increase in sensitivity between 2D-TVS and 3D-TVS from 79.5% to 90.5% and a decrease in specificity from 89.6 to 84.3% is observed in terms of the evaluation of deep myometrial invasion in endometrial cancer patients [38]. Another study assessed the value of 3D-TVS in detecting abnormally positioned IUDs in patients presenting with pelvic pain and AUB. The retrospective analysis included 167 women with the presence of an IUD in their uteri noticed on 2D-TVS. In 28 of them (28/167), the malposition of the IUD was detected with the 3D-TVS, with the uterus viewed in a coronal plane; however, it was not noticed in the regular 2D-TVS, in which only typical views of the shaft of the IUD could be seen. It is suggested that the coronal view of the uterus obtained by 3D-TVS allows the visualization of an entire IUD and helps identify the cause of bleeding or pelvic pain, possibly caused by the malpositioned IUD [37].

A different analysis demonstrated whether 3D-TVS obtained any additional information on endometrium and adjacent myometrium compared to the result of prior 2D-TVS imaging among 90 randomly selected patients. During both examinations, sonographers analyzed the endometrium and myometrium for the architecture and anatomical aspects of the uterine cavity, abnormalities such as masses, and their relationship to the endometrial cavity. Additional information obtained with the coronal view of 3D-TVS was found in 28 cases (30.8%, 28 of 91 examinations). Among these 28, the additional information concerned a uterine anomaly (8/28), better definition of the endometrium (7/28), delineation and location of polyps (6/28), location of leiomyomas (3/28), and confirming IUD location (2/28). In 3 out of 7 cases of better delineation of endometrium with 3D-TVS, the 2D-TVS scan visualized the endometrium poorly due to adenomyosis [38]. 

The coronal plane of the uterus obtained with 3D-TVS, which is rarely seen in 2D imaging, often provides additional information to the regular TVS, allowing for delineation of gynecological pathologies. It enables the viewing of the entire length of the endometrial cavity and the cervix [31]. It gives details on the orientation of the uterus within the pelvis. As with 3D-TVS, any “desired” plane can be obtained due to the multiplanar reconstruction, in which the role of the initial orientation of the beam is reduced [33]; perhaps it may overcome the limitations of 2D-TVS concerning unfavorable uterine positions. They originate from the inadequate angle of insonation in certain uterine positions, such as the axial position. Considering the above, the 3D-TVS might be a valuable tool which could extend the possibilities of the regular TVS, particularly in the assessment of the uteri of less frequent positions (Table 2).

## 6. Transabdominal Ultrasound

TAS, alongside TVS, are the two foundational screening tools in gynecological imaging [40]. The efficacy of TAS is strictly dependent on the presence of a full urinary bladder, which creates an “acoustic window” [41] by displacing bowel loops and providing a reference point for pelvic anatomy. While bladder distention affects the orientation of the anatomy, the lower spatial resolution remains a primary limiting factor for transabdominal assessment of this region. In clinical practice, a full bladder causes a patient’s discomfort and [13] can induce an “anteverting effect”. This effect can be explained as a mechanical displacement caused by the pressure exerted by the filled bladder on the uterine fundus, masking uterine retroflexion by pushing the fundus forward. Therefore, while a full bladder is necessary for TAS, it can paradoxically distort the very anatomy it aims to visualize. Despite aforementioned limitations, the primary advantage of TAS is its non-invasiveness and painlessness; thus, it is the preferred diagnostic tool for pediatric patients and sexually inactive women [42]. This approach is also considered the safest and most effective diagnostic route in early puerperium [43]. Additionally, during the first weeks postpartum, the uterus is significantly enlarged and remains in close contact with the anterior abdominal wall, often naturally displacing bowel loops. This allows for a comprehensive uterine assessment without the need for invasive transvaginal probes, which is particularly beneficial for patients who experienced obstetric trauma [43]. 

Furthermore, TAS is indispensable for providing a ‘global overview’ of the pelvis, offering superior visualization of large leiomyomas or adnexal masses that may exceed the focal range of transvaginal probes [44]. In addition to mapping large structures, research indicates that TAS is highly effective for specific localized tasks, such as confirming the correct position of IUDs or monitoring their potential displacement [45]. In conclusion, the broader field of view provided by TAS ensures clear visualization of the relationship between the uterus and its surrounding pelvic structures, even in the presence of significant uterine enlargement or displacement. The reliability of TAS in measuring ET and identifying pathologies is significantly influenced by uterine orientation and patient anatomy. A cross-sectional study of 50 postmenopausal women demonstrated a systematic tendency of TAS to underestimate measurements, reporting a mean ET of 4.46 ± 3.05 mm compared to 5.28 ± 3.83 mm via TVS (*p* < 0.001) [4]. Importantly, compared with the endometrial biopsy—the diagnostic gold standard—TAS results did not correlate with histopathological examination in 34% of cases (17 out of 50 patients), whereas TVS showed a significantly higher correlation rate [4] These diagnostic discrepancies are primarily attributed to the inherent limitations of the transabdominal approach in visualizing pelvic organs, particularly when they are atrophic or poorly positioned [4].

The most significant technical barrier for TAS is uterine retroversion and retroflexion. The difference in mean ET measurements between TAS and TVS is larger in patients with a retroflexed uterus than in those with an anteflexed one [8]. This is further supported by another article, where in cases of a retroverted or retroflexed uterus, TAS did not visualize the endometrial canal echo in 73% of instances (16 out of 22 studies) in contrast to TVS, which was successful in 100% of the same cases [44]. This imaging difficulty is due to the fact that in the retroverted position the uterine fundus is often outside the optimal focal zone of the transducer. These results align with yet another article, which reported that TAS failed to provide a clear view of the endometrium in 16 out of 90 cases, identifying retroversion (8 patients), fibroids (3 patients), and prolapsed uterus (5 patients) as the main reasons for failure. The inability of TAS to clearly resolve the endometrial canal can lead to missed focal lesions or the misinterpretation of uterine pathology. This highlights the need to perform TVS in cases where the uterus is not anteflexed [46].

The axial or mid-position uterus presents a unique diagnostic trap in TAS where imaging failure is often more closely related to the loss of acoustic optimization than to the angle of insonation alone. One of the primary factors is the lower frequency of TAS probes, typically ranging from 3.5 to 5 MHz, which is necessary to achieve sufficient depth but inherently results in lower spatial resolution [47,48]. This makes it difficult to distinguish the endometrial-myometrial interface, which clinically manifests as blurred borders. The vertical orientation of an axial uterus forces the ultrasound beam to travel through intervening soft tissues and bowel loops, resulting in loss of the acoustic window. This path increases beam scattering and attenuation, significantly degrading the signal quality [26]. The mandatory use of lower frequencies (3.5–7 MHz) in transabdominal scanning to achieve adequate penetration depth further compromises spatial resolution, increasing the loss of anatomical detail [49]. Because the axial uterus is situated at a greater depth, the signal strength is significantly reduced, causing the endometrial borders to appear blurry. While authors [46] focused on TAS in general, their findings confirm that such limitations in image clarity often led to an artificial overestimation of ET and a higher rate of false-positive findings [46].

Diagnostic accuracy is further compromised by patient-specific factors and the inherent physical limitations of the transabdominal approach. A study of 100 women demonstrated that TAS has a significantly lower specificity (68.4%) than TVS; they noted that the method is particularly ineffective at resolving focal lesions, such as polyps, which were detected by TAS in only 2 out of 9 confirmed in histopathology cases [50]. This shows that the presence of deformation in the myometrium affects the visibility of the endometrial canal. Furthermore, technical factors such as increased BMI and abundant abdominal adipose tissue contribute to significant beam scattering and attenuation [46]. When these factors coexist with an axial or retroverted position, the diagnostic yield of TAS drops significantly as the signal must travel through thicker, attenuating tissues without the benefit of a clear acoustic window. Table 3 presents the main characteristics of studies assessing TAS in the context of the uterine position. 

In conclusion, while TAS remains a valuable and painless “mapping” tool for a global overview of the pelvis, it possesses inherent diagnostic blind spots. The technical limitations of TAS in retroverted or axial uteri—that result from lower spatial resolution, the loss of the bladder’s acoustic window, and the physics of beam attenuation at depth—necessitate the complementary use of TVS to resolve diagnostic ambiguity and prevent clinical errors in endometrial assessment.

## 7. Transrectal Ultrasound

TRS is used in gynecology primarily in patients in whom TVS cannot be performed, such as sexually inactive women, pregnant women with suspected cervical insufficiency, or postmenopausal women with severe vaginal atrophy [51]. In addition, TRS may serve as an adjunctive imaging modality during transvaginal surgical procedures by providing real-time guidance [52]. Furthermore, the available literature indicates several clinical situations in which gynecological patients may benefit more from pelvic imaging with TRS than with TVS [3]. These include imperforate hymen, other vaginal pathologies, endometriosis, and, as it turns out, axial uterus [3,53,54]. In patients with an axial uterus, during standard TVS, the ultrasound beam does not fall at a 90° angle to the long axis of the endometrium, which may compromise the quality of visualization of the endometrium and uterine cavity. Furthermore, in the axial uterus, the ultrasound beam is parallel to the endometrium, which can produce a false, three-layer echo pattern and, therefore, a falsely hyperechoic result [29]. During TRS, the transducer is positioned at the level of the internal cervical os. Furthermore, compressing the uterine fundus allows for uterine retroflexion, which may lead to improved image visualization in women with an axial uterus [3].

One study aimed to assess the acceptability and efficacy of TRS compared to TVS in 103 patients with an axial uterus, including 50 patients with PMB [3]. Both TVS and TRS were performed during the same visit. Interestingly, univariate analysis showed that patients with an axial uterus were more likely to be nonparturient than those with a retroverted or anteverted uterus. TVS failed to adequately assess the endometrium in 15/50 patients with PMB compared with TRS, which assessed the endometrium satisfactorily in all of these patients. Furthermore, in 35 patients whose endometrium was visualized by both examinations, ET was thinner by TRS than by TVS (median difference—1.2). Importantly, 7 women were diagnosed with endometrial cancer by TRS, 4 of whom were initially diagnosed as benign polyps by TVS [3]. Given the above, it is important to note that in women with an axial uterus, certain features suggestive of endometrial malignancy may be more easily diagnosed by TRS. This is important for an appropriate qualification for further invasive tests such as endometrial biopsy or hysteroscopy, which may be avoided in patients additionally evaluated with TRS.

Interestingly, it turns out that the authors of another study had previously pointed to the axial uterus as a limitation in visualizing the endometrium and uterine cavity [5]. There were 472 postmenopausal patients in whose case TVS was performed to assess ET. If the endometrium could not be visualized, the reason was noted. In 180 patients, the endometrium was not satisfactorily visualized. The most common causes for inadequate assessment quality were fibroids (20.1%), adenomyosis (7.4%), and axial uterus (10.6%) [5].

The most recent study evaluated satisfactory ET assessments using TVS compared to a multimodal sonographic approach (TVS, TRS and TAS) in 387 women with AUB [27]. When the endometrium could not be visualized with TVS, women were assessed using an alternative approach—TAS, TRS, or SCSH. Unfavorable uterine position accounted for 26.1% (37 of 142 patients) of the causes of suboptimal TVS results. The authors of this study confirmed the results of Wong et al.—ET measurement was higher with TVS compared to a multimodal sonographic approach (median difference—1.3) [3].

Another study compared TRS with TVS, but only among women with AUB. The aim of the study was to evaluate and compare the diagnostic accuracy of TRS with TVS in women with AUB. Forty patients included in the study underwent both TRS and TVS, with endometrial biopsy as the gold standard. The results obtained using these tests were found to be consistent with each other regarding characteristics such as uterine size, endomyometrial junction, myometrial homogeneity, uterine lesions, uterine lesion size, ovarian size, and ovarian echogenicity. In contrast to the results of the first study [3], in this study, ET did not differ between the two methods (8.01 ± 5.3 mm in the transvaginal method and 7.95 ± 5.2 mm in the transrectal method). Regarding uterine position, the uterus was retroverted in 4 patients, anteverted in 18, and normal in 18 [55]. 

Another study also demonstrated that TRS is superior to TVS in patients with a retroverted uterus. In such patients, the endometrial stripe is virtually parallel to the ultrasound beam and cannot be accurately measured with TVS [56]. However, the study results were based on only 10 women; therefore, further research is needed to assess the diagnostic accuracy of TRS and TVS, but in a larger group of patients.

Considering the above, TRS has a clear advantage over the commonly used TVS for endometrial imaging in patients with an axial or retroverted uterus. The use of TRS may potentially improve ET assessment and thus prevent unnecessary referrals for biopsy or hysteroscopy. This is clinically relevant not only from the patient’s perspective, by minimizing anxiety and discomfort associated with invasive procedures, but also from a healthcare system perspective, as it may contribute to a reduction in hospital-related costs and unnecessary interventions. Perhaps the more frequent use of TRS in this patient group will allow for the development of new treatment algorithms in the future, where the choice of method for visualizing the endometrium and uterine cavity will also depend on the position of the uterus (Table 4).

## 8. Discussion

This is the first narrative review supported by systematic search of the literature to summarize the evidence on diagnostic accuracy of different ultrasound approaches for assessment of uterine cavity pathologies. Despite numerous studies describing the limitations of uterine ultrasound imaging, the impact of uterine position remains unclear. Our narrative review highlights a substantial gap in the literature regarding the impact of uterine position on the visualization of intrauterine lesions and the accuracy of ET assessment. In particular, studies investigating the mechanisms by which specific uterine positions may impair or alter ultrasonographic evaluation remain notably scarce. 

TVS still remains the first-line imaging in gynecological practice due to its accessibility, high resolution, and diagnostic performance in the assessment of endometrial and myometrial pathology. Our narrative review only confirms its value in the evaluation of ET, as it is a crucial and necessary tool for identifying patients at increased risk of endometrial malignancy.

However, the available evidence reviewed by us emphasizes the limitations of TVS. Uterine position, such as axial and retroverted uteri, can significantly compromise visualization of the endometrium and the accuracy of ET measurements. Hence, the presence of the axial uterus may potentially lead to delayed diagnosis of endometrial hyperplasia, endometrial cancer, or endometrial polyps [5]. 

Moreover, retroversion and retroflexion may be inconsistently assessed depending on bladder filling status and probe manipulation, which may lead to misclassification of uterine orientation. One study mentions a limitation of the TVS in the diagnosis of retroflexion in women with endometriosis [30]. This analysis shows superiority of MRI over TVS as it provides a more objective assessment of the position of the uterus. MRI is, however, associated with higher costs, lower availability, and the average examination time is longer than in TVS.

Abnormal uterine version and flexion may not only influence the assessment of uterine orientation itself but can also affect the ultrasonographic evaluation of other gynecologic diseases. An axial uterus prevents obtaining an optimal insonation angle and thus may impair the visualization of endometrium and intrauterine lesions in TVS [6]. Extreme retroflexion and other atypical positions can limit the viewing of the uterine cavity in 3D-TVS [57], an important tool in assessing congenital uterine diseases. Furthermore, a common finding among women with deep infiltrating endometriosis is a retroverted or retroflexed uterus caused by adhesions. International radiological recommendations prioritize the evaluation of uterine position, organ mobility, and the sliding sign during TVS, as these features are important for recognizing the posterior compartment disease. Therefore, altered uterine position and reduced mobility may be a limitation of TVS and pose a difficulty in objective image interpretation [58,59].

What is important, available evidence emphasizes that failure to adequately visualize the endometrium should not be interpreted as reassuring [5]. In such cases, reliance solely on TVS may lead to underdiagnosis of endometrial pathology. Complementary imaging modalities, such as SCSH hysteroscopy or MRI, may be considered, particularly in suspicious cases. When these options are not available due to long waiting times or cost, gynecologists and clinicians should take other visualizing options as well into account—such as TRS or TAS [27]. 

The study comparing TRS with TVS in postmenopausal patients with an axial uterus [3], as well as the conclusions drawn by the other study [27], highlight the need to identify which patients are most likely to benefit from TRS. TRS, although not a commonly used method by every clinician in gynecological patients, has been shown to have significant benefits in patients with an axial or retroverted uterus. Overestimating the ET in patients with an axial uterus using TVS can lead to unnecessary biopsies or hysteroscopy, which therefore poses a psychological burden and unnecessary stress for women. TRS, as an alternative, can serve as an additional method, complementing TVS and excluding thickened endometrium in women with an axial uterus. Using this method has another advantage: it is inexpensive and carries a lower risk of complications.

However, the study comparing TRS with TVS in postmenopausal patients with an axial uterus has several limitations. First, both TVS and TRS were performed by the same operator, which made blinding difficult. Second, such a study needs to be conducted on a larger number of patients. Axial uterus is rare—it occurs in approximately 5% of patients [10], which makes it difficult to obtain a sufficient sample of women for examination. Last but not least, the pressure exerted by the transducer during the examination may vary depending on the operator, which may also affect not only the examination results but also the position of the uterus and the angle between the cervical axis and the endometrium. This variability generates the need for a multicenter study to confirm the validity of the findings as well as intra- and interobserver reliability. Furthermore, sonographers should be experts in gynecological ultrasound with years of experience. Finally, it is crucial that every clinician performs the examination according to this protocol. The guidelines proposed by IETA may be helpful in this regard [6]. 

Another study emphasizes the importance of using multiple imaging modalities to better visualize the endometrium, but it has limitations. The authors did not assess the influence of individual or patient-specific factors, such as the use of an IUD or hormone replacement therapy (HRT). Their impact on endometrial assessment should be further explored in future studies [27]. 

2D-TVS is the first imaging modality due to its widespread use, low cost, and high diagnostic accuracy in most patients. However, 3D-TVS can provide additional information through coronal plane reconstruction, which is of great value in diagnosing uterine morphology and congenital anomalies, while also being less expensive than MRI. In patients with contraindications to TVS, TRS may be helpful, especially in patients with an axial uterus. Although TAS provides a broader view of the pelvis, such as in cases of large uterine lesions, it has lower spatial resolution.

While the goal of our narrative review is to compare different ultrasound modalities in the assessment of uterine pathology depending on uterine location, it remains important that MRI remains the gold standard for assessing soft tissue. Furthermore, it is the reference imaging modality in patients with adenomyosis or complex Müllerian anomalies. Another diagnostic method, hysteroscopy, allows for direct assessment and diagnosis of the uterine cavity while simultaneously treating lesions. CT appears to have a limited role due to its poorer assessment of soft tissue and exposure to ionizing radiation [60,61]. Importantly, despite the advantages of methods such as MRI and hysteroscopy, these techniques are less accessible, more expensive, and invasive, and are therefore used as adjunctive techniques in selected patients.

However, pain remains an important factor influencing patient consent to a transrectal examination. Wachsberg et al. noted that patients consented to TRS when informed that the examination was necessary for diagnostic purposes and to prevent other unnecessary interventions [56]. In the study [3], only 1 in 66 patients had a TRS examination interrupted due to pain [3]. Pain associated with TRS can be prevented by pre-treatment with lidocaine gel. However, another study on the Korean population did not find that the use of lidocaine gel reduced pain in patients compared to a group of patients treated with a standard lubricant gel [62]. Therefore, informing and educating patients about the benefits of additional TRS in addition to TVS in gynecological diagnostics seems important, especially when the woman has factors that complicate TVS, such as a retroverted or axial uterus.

From a clinical perspective, the results of our narrative review indicate that the choice of ultrasound method for diagnosing uterine anomalies in women can be guided by uterine position. Available evidence, although limited, suggests that TRS may be an alternative imaging technique when TVS is insufficient in women with an axial or retroverted uterus. However, the development of a standardized diagnostic algorithm is not possible due to the limited number of currently available studies and their methodological heterogeneity. Therefore, further prospective, multicenter studies are crucial to determine the appropriate sequence of imaging methods for different clinical situations. These studies should assess the impact of uterine position on the diagnostic accuracy of specific imaging methods in both premenopausal and postmenopausal women. Therefore, standardized imaging protocols, as well as interobserver agreement and patient acceptability assessment, are essential, which will provide new evidence (Table 5).

## 9. Conclusions

Satisfactory visualization of the uterus irrespective of uterine position appears to require an individualized diagnostic approach that incorporates more than a single imaging modality. Although TVS remains the first-line imaging technique, the available evidence suggests that TAS and TRS may provide substantial additional value in patients with an axial or retroverted uterus. In particular, the use of TRS may reduce false-positive ET measurements and consequently decrease the number of unnecessary invasive procedures, such as endometrial biopsy or hysteroscopy. Moreover, TRS may serve as a valuable alternative when SCSH is unavailable or contraindicated.

Literature regarding the influence of uterine position on the visualization of the endometrium and intrauterine lesions is scarce. Available studies are further constrained by relatively small sample sizes, heterogeneous study populations, and the absence of blinded assessment. Therefore, future large-scale, multicenter studies are warranted to evaluate intra- and interobserver reproducibility and to better define the clinical indications for the use of TRS. This is of particular importance, as TRS may emerge in the future as an adjunctive or alternative diagnostic modality in the evaluation of endometrial pathology, including endometrial cancer.

## Figures and Tables

**Table 1:** Main characteristics of studies assessing TVS regardless of uterine position.

Author of the Study	Year of the Study	Research Group	Uterus Location	Main Findings
Goldstein et al. [5]	2021	472 asymptomatic postmenopausal women	50 women with an axial uterus.	180 (38.1%) women had anatomical reasons for suboptimal visualization of the endometrium with TVS and lack of adequate ET measurement. In 50 of the women involved the reason was the presence of the axial uterus.
Sanders et al. [10]	2014	641 women; patientswere further dividedinto 3 groups: thosewho underwentcesarean delivery (CD), patients whowere parous but hadnot undergone CD and nulliparous patients	499 (77.8%) patients with an anteverted uterus, 77 (12%) patients with a retroverted uterus, 32 patients with anaxial uterus (5%), 31 (4.8%) patients with antevertedretroflexed, 2 (0.3%) patients with retroverted anteflexed uterus	32% of retroverted uteri were apparently anteverted at the abdominal examination but werer etroverted when the transvaginal probe was used.
Karlsson et al. [28]	1995	1168 women with PMB scheduled for curettage	–	In 30 of 1168 (2.8%) patients the measurement of endometrium could not be obtained due to difficulties in identifying the longitudinal echo line
Haylen et al. [13]	2007	480 women of reproductive and menopausal age	In TVS: 86 (18%) patients with retroverted uterus, The study did not include information concerning uterine position of the remaining women.	A full bladder tends to antvert the uterus masking its natural orientation; in TAS the prevalence of retroversion was 13% and in TVS it was 18% (86/480)
Matsuda et al. [30]	2023	123 women who underwent endometriosis surgery	TVS: 71 cases of anteflexion and 52 of retroflexion MRI: 43 cases of anteflexion, 80 of retroflexion 67/123 patients with anteverted uterus and 56/123 patients with a retroverted uterus.	TVS correctly diagnosed 43 cases of anteflexion and misdiagnosed 28 retroflexed uteri as anteflexed, the 28 misdiagnoses concerned the anteverted uteri suggesting a limitation of TVS in the diagnosis of retroflexion in women with endometriosis

**Table 2:** Main characteristics of studies assessing 3D-TVS.

Author of the Study	Year of the Study	Research Group	Uterus Location	Main Findings
Benacerraf et al. [37]	2009	167 consecutive patients with an IUD	–	A coronal view of the uterus allows the visualization of an entire IUD and helps identify the cause of bleeding or pelvic pain, possibly being the malpositioned IUD
Andreotti et al. [38]	2006	90 randomly selected patients	–	Of 91 studies (2D-TVS and further 3D-TVS) 28 studiesin the coronal plane with 3D-TVS revealed additionalf indings
Sakhel et al. [39]	2014	51 patients with a normal uterus	33 patients (64.7%) with an anteverted uterus, 13 (25.5%) patients with a retroverted uterus, 5 (9.8%) with an axial uterus	The uterus can be rotated at the fundus a horizontal plane

**Table 3:** Main characteristics of studies assessing TAS in the context of the uterine position.

Author of the Study	Year of the Study	Research Group	Uterus Location	Main Findings
Haylen BT [13]	2007	480 women of reproductive and menopausal age underwent TAS (full bladder) and TVS scanning (empty bladder). None were pregnant or recently pregnant.	In TVS: 86 (18%) patientswith retroverted uterus.	The full bladder required for TAS causes an anteverting effect, which results in a reduced detection of retroverted uterus.
Mulic-Lutvicaet al. [43]	2001	42 women with uncomplicated vaginalterm deliveries wereexamined serially by ultrasound on postpartum days 1, 3, 7,14, 28 and 56.	–	During the first 14 days of postpartum, TAS is adequate for uterine evaluation. After day 28 TVS is recommended.
Coleman et al. [44]	1988	215 patients (aged 14–80) were divided into 6 categories, includinggroup 4 (retroverted/retroflexed uterus)	22 patients with retroflexed orretroverted uterus.	TAS showed retroflexion or retroversion of uterus in 6 of 22 cases, in contrast to TVS which showed 22 of 22
Palo et al. [45]	1997	20 fertile women with normal sized uteri were evaluated via TAS and TVS using 3different ultrasound devices. Examinationswere conducted beforeand after the placement of a levonorgestrel-releasing IUD.	–	Both TAS and TVS are effective for locating a levonorgestrel-releasing IUD. Both methods allow for clear visualization of the device’s ends and confirm its correct placement within the uterine fundus.
Morampudi et al. [4]	2024	50 women of menopausal age	Anteflexed and retroflexed	TAS showed little correlation with histopathological results and significantly underestimated ET, in contrast to TVS which correlated with histopathological results
Tsuda et al. [8]	1995	91 postmenopausal women	Anteflexed *(n* = 74) Retroflexed (*n* = 17)	Retroflexed uterus causes greater differences observedin measurements obtained by TAS and TVS.
Nasri et al. [46]	1991	90 women underwentTAS 111 women underwentTVS	Retroflexed (*n* = 8) in TAS Retroflexed in TVS not specified	Retroflexed uterus (*n* = 8) significantly impaired long-axis endometrial visualization in TAS. Similar difficulties were observed with uterine prolapse (*n* = 19), where measurement was impossible or unreliable in 5 patients. The presence of fibroids (*n* = 3) completelyo bscured the endometrial line in TAS. TVS was effective in all of the above cases, also demonstrating resistance to obesity-related artifacts which made TAS diagnosis difficult.
Sedeq et al. [50]	2016	group of 100 women with AUB was evaluated: 74 were in the pre-menopausal stage, while 26 were post-menopausal.	–	The low specificity of TAS (68.4%) and difficulties in imaging the endometrial line in fibroids result in poordetection of focal lesions, including polyps.

**Table 4:** Main characteristics of studies comparing TRS with TVS.

Author of the Study	Year of the Study	Research Group	Uterus Location	Main Findings
Wong et al. [3]	2022	103 patients with axialuterus, of whom 76 presented with PMB.	103 patients with axial uterus	TRS may be associated with more satisfactory endometrial assessments and fewer misdiagnoses of endometrial cancerc ompared with TVS.
Nijjar et al. [27]	2026	387 women	37 patients with “unfavorableuterine position”	Compared with TVS alone, the MM approach mayallow more accuratev isualization of the endometrium in women with AUB. Furthermore, it improved endometri alassessment through betteroptimization of the insonation angle and clearer depiction of the uterine cavity, leading to significantly lower ET values.
Akbari Sene et al. [55]	2022	40 patients	antroverted uterus in 18 patients retroverted uterus in 4 patients normal uterus in 18 patients	The agreement between the findings of TVS and TRS for uterine size, ET and uterine abnormalities was high.
Wachsberg et al. [56]	2003	10 patients (case seriesstudy)	Retroverted uterus in 1 patient with PMB.	TRS was necessary in all women with a retroverted uterus due to the fact that the endometrial stripe was virtually parallel to the ultrasound beam.

**Table 5:** Advantages and limitations of different ultrasound modalities according to uterine position [5,8,13,21,31,44].

Ultrasound Modality	Anteverted Uterus	Axial Uterus	Retroverted Uterus	Main Advantages	Main Limitations
2D-TVS	Excellent visualization; first-line diagnostic method.	The quality of image may be reduced due to parallel insonation of the endometrium.	Often adequate, however visualization may occasionally be limited.	High availability, inexpensive and real-time imaging method.	Operator-dependent; reduced diagnostic performance in selected uterine positions (axial, retroverted uteri).
3D-TVS	Very good assessment of uterine morphology and cavity.	The possibility of coronal reconstruction may lead to improvement of assessment compared with 2D -TVS but image acquisition may remain technically challenging.	Good visualization.	Superior evaluation of congenital anomalies and uterine cavity in the coronal reconstruction.	Dedicated equipment and expertise is needed.
TAS	Very good overview of the whole pelvis.	May be a complement diagnostic method to TVS/TRS, particularly in large uteri.	Useful for global pelvic assessment.	Wide field of view; non-invasive.	Lower spatial resolution as well as limited assessment of endometrium and intracavitary lesions.
TRS	Not a first line of diagnostic modality in these uteri.	May be useful when TVS is technically limited by axial uterine position or contraindications to TVS exist.	Useful alternative when TVS is not feasible.	High-resolution imaging with no vaginal access.	Limited availability and operator experience, patients’ concerns about this method.

## Data Availability

No new data were created or analyzed in this study. Data sharing is not applicable to this article.

## Abbreviations

The following abbreviations are used in this manuscript:

TVS	Transvaginal ultrasound
SCSH	Saline contrast sonohysterography
TAS	Transabdominal ultrasound
TRS	Transrectal ultrasound
IUD	Intrauterine device
PMB	Postmenopausal bleeding
ET	Endometrial thickness
3D-TVS	Three-dimensional transvaginal ultrasound
JZ	Junctional Zone
AUB	Abnormal uterine bleeding
HRT	Hormonal replacement therapy
